# Lung clearance index to detect the efficacy of Aztreonam lysine inhalation in patients with cystic fibrosis and near normal spirometry – A single-centre feasibility study

**DOI:** 10.1371/journal.pone.0221673

**Published:** 2019-09-09

**Authors:** Helmut Ellemunter, Gratiana Steinkamp

**Affiliations:** 1 Cystic Fibrosis (CF) Centre at the Medical University of Innsbruck, Innsbruck, Austria; 2 Clinical Research and Medical Scientific Writing, Schwerin, Germany; Public Library of Science, UNITED KINGDOM

## Abstract

Comparing the efficacy of inhaled antibiotics can be difficult in small groups of patients with cystic fibrosis and mild lung disease. In a feasibility study we compared Aztreonam lysine for inhalation solution (AZLI; Cayston^®^) to standard inhaled antibiotic therapy in patients with cystic fibrosis and near normal spirometry_._ To detect treatment responses we used both lung clearance index (LCI) and forced expiratory volume in one second (FEV_1_). At baseline, median FEV_1_ was 87% pred. and median LCI was 8.6 (upper limit of normal: 7.0). After 4 weeks, LCI improved by -0.36 after AZLI and deteriorated by +0.12 after tobramycin treatment (p = 0.039). No significant differences between treatments (p = 0.195) were observed using FEV_1_. These results suggest that lung clearance index can be used to detect treatment induced changes in subjects with mild lung disease.

## Introduction

Inhaled antibiotics are important for the suppression of chronic *P*. *aeruginosa* (PA) infection in patients with cystic fibrosis (CF). Aztreonam lysine (AZLI, Cayston^®^) for inhalation solution was licensed in Europe in 2009 for the suppressive therapy of chronic pulmonary infections due to *Pseudomonas aeruginosa* in patients with CF aged 6 years and older. The pivotal clinical trials were conducted in patients with impaired lung function, i.e. forced expiratory volume in one second (FEV_1_) ≤75% of the predicted normal value[1–3]. In patients with moderate to severe lung disease, Aztreonam was superior to tobramycin inhalation.

Due to the improved general condition of current CF populations, many patients have normal or near normal FEV_1_ despite chronic *P*. *aeruginosa* infection [4]. Only little information is available about the efficacy of inhaled antipseudomonal antibiotics in these subjects. In general, with small patient cohorts of up to 20 patients, it is difficult to detect changes after treatment using FEV_1_ as an endpoint [5,6]. This is particularly relevant if two active compounds are to be compared or if the inhaled antibiotic is switched from a conventional to a new drug. At the CF Centre Innsbruck, we have been using the multiple breath washout (MBW) technique for many years to measure lung clearance index (LCI) [7]. Abnormal LCI is associated with early structural lung disease detectable by high resolution computerized tomography (HRCT) scans when FEV_1_ may still be normal.

For the present feasibility study in subjects with mild lung disease, we hypothesised that LCI could better detect improvements after inhaled antibiotics than FEV_1_. When the study was designed, AZLI was not yet a standard treatment at our centre. At that time, patients with chronic *P*. *aeruginosa* infection inhaled either tobramycin or colistin on a long-term basis. We performed a pilot study comparing AZLI with standard inhaled antibiotics in a small group of patients with normal FEV_1_ (≥75% of the predicted normal value).

## Methods

This single-centre, observational, open-label, feasibility study compared two treatment phases, each consisting of 4-week on/off-cycles with inhaled antibiotics: Phase 1, weeks 0 to 8: standard inhaled antibiotic (tobramycin/TOBI^®^ 300mg/5ml BID or TOBI Podhaler^®^ 112mg BID), and Phase 2, weeks 8 to 16: AZLI 75 mg TID. ALZI was provided by Gilead Sciences, the manufacturer of AZLI. For each patient, the study started at the end of a 4-week off-period without standard inhaled antibiotic (“washout”). The patient recruitment period was from June 2014 to January 2016.

The main inclusion criteria were: clinically stable patients aged ≥12 years with CF, FEV_1_ ≥75% of the predicted normal value, chronic *P*. *aeruginosa* lung infection [8], and at least two previous on/off cycles or > 8 weeks of continuous inhaled antibiotic treatment with tobramycin. Major exclusion criteria were age ≥ 50 years, acute upper or lower respiratory infections or pulmonary exacerbations.

The primary endpoint was lung clearance index (LCI) measured by nitrogen multiple breath washout using 100% oxygen (EasyOne Pro^®^ LAB MBW Module, ndd Medical Technologies, Zürich, Switzerland), with an upper limit of normal of 7.0 [9]. MBW testing and spirometry were performed before a session of standard physiotherapy. Secondary endpoints were prebronchodilator FEV_1_ using reference values from the global lung initiative (GLI) [10]. Respiratory symptoms were determined with the Cystic Fibrosis Questionnaire—Revised Respiratory Symptom scale [11]. Adverse events were documented at each study visit.

When we initially designed the study, we performed a sample size calculation. During our five years of experience with LCI measurements we had observed a LCI standard deviation of 1.25 in relatively healthy CF patients. For the present study, we assumed that LCI will improve after AZLI by at least the same magnitude as after hypertonic saline [12], probably more. Using these figures, a sample size of 10 had 80% power to detect a difference between means of 0.85 with a significance level (alpha) of 0.05 (two-tailed) in a paired t-test.

Data are expressed as median and range unless otherwise stated. For each parameter, the relative changes from start to end of the two treatment periods were determined. The non-parametric Wilcoxon signed rank test was used to compare the relative changes during AZLI and standard treatment phases. The study protocol (S1 Protocol) was approved by the Ethics committee of Medical University of Innsbruck (EC Number: UN 2013–0014_LEK Sitzungsnummer: 330/2.5), EudraCT Number: 2013-004295-35, there was no deviation from this study protocol (S1 Protocol). Written informed consent was obtained from all individual participants included in the study.

## Results

**Fig 1:**
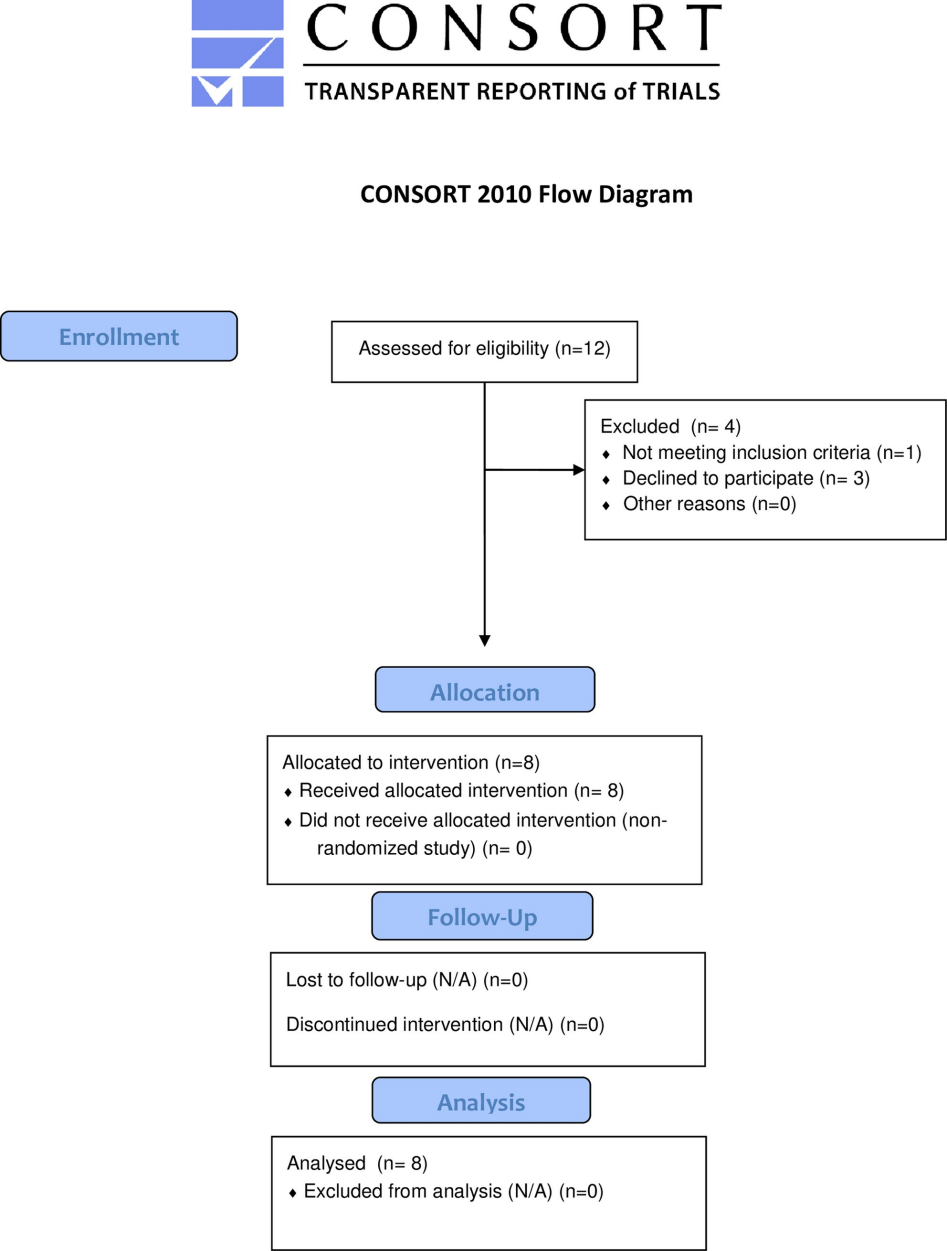
CONSORT participant flow diagram.

A total of 12 patients were approached, three patients had too little time to participate in the study due to heavy workload, and one subject did not meet inclusion criteria. Eight patients with CF aged 15 to 49 years (median 28 yrs.) participated in the trial (Fig 1). They had been chronically infected with *P*. *aeruginosa* for 6.5 years and had inhaled antipseudomonal antibiotics for 4.2 years. FEV_1_ ranged from 76.3 to 123.8% predicted (median 87.0%) and the FEV_1_ GLI z-score from -2.20 to 2.04 (median -1.24). Median body mass index was normal (21.5 kg/m^2^), and the median CFQ-R Respiratory symptom scale of 83.3 (range 78 to 94) showed only few limitations. Despite near normal spirometry, the median lung clearance index of 8.59 (range 6.4 to 11.4) was above the upper limit of normal (7.0), with only two patients showing normal values at the start of the study. The standard inhaled antibiotic was tobramycin in all subjects, with n = 5 subjects using TOBI Podhaler^®^ 112 mg BID and n = 3 inhaling TOBI^®^ 300 mg/5 ml nebuliser solution BID. Since previous authors had reported comparable safety and efficacy profiles of the two tobramycin treatments [13,14], we analysed the combined results from both treatments.

**Fig 2:**
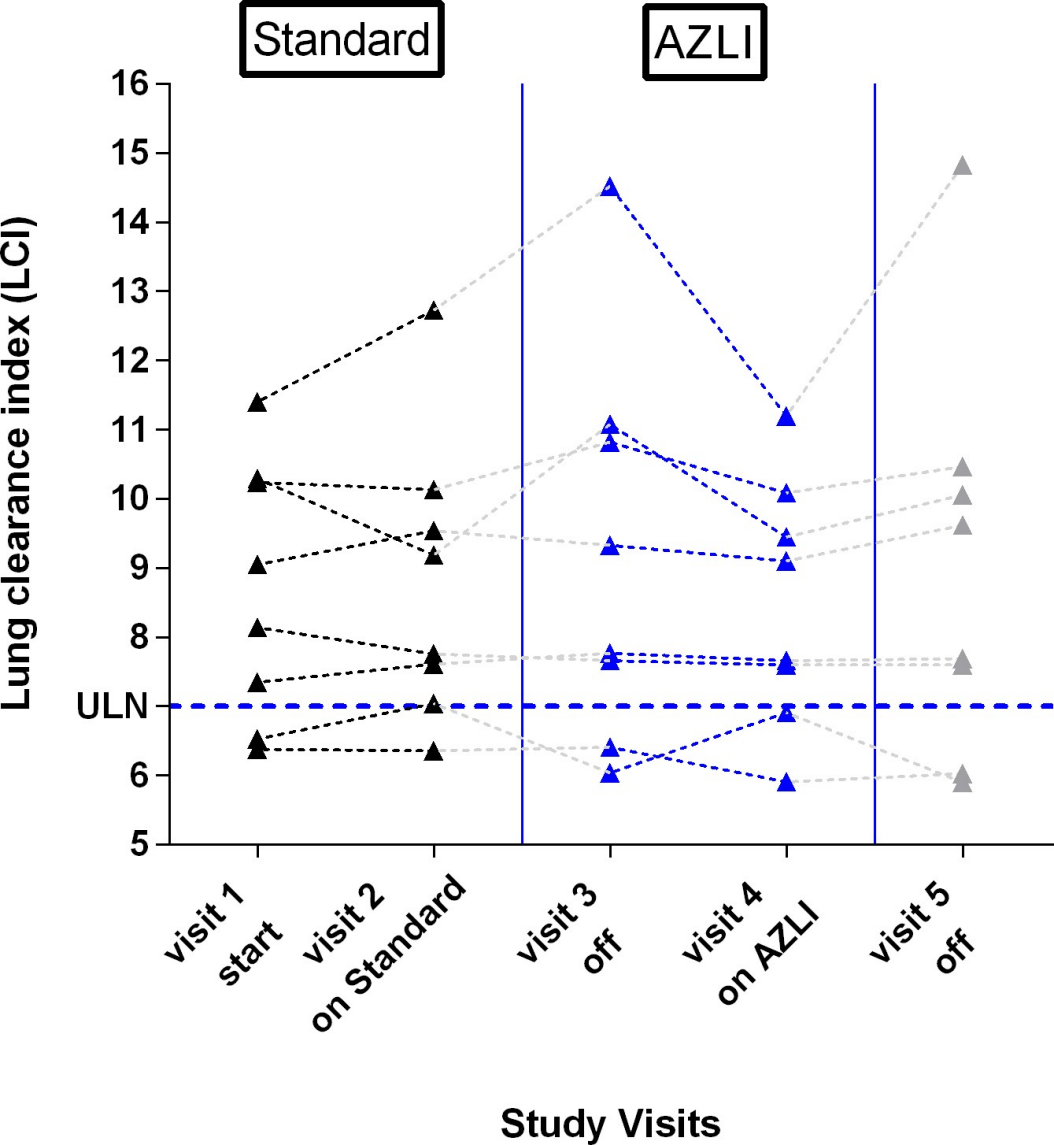
LCI during and after inhalation with TOBI^®^ and AZLI. Decreasing values indicate improvement in lung function.

After 4 weeks of AZLI treatment, the primary endpoint LCI improved (i.e. declined) in 7 of 8 patients, while only 4 of 8 patients showed better results after tobramycin inhalation (Fig 2).

**Fig 3:**
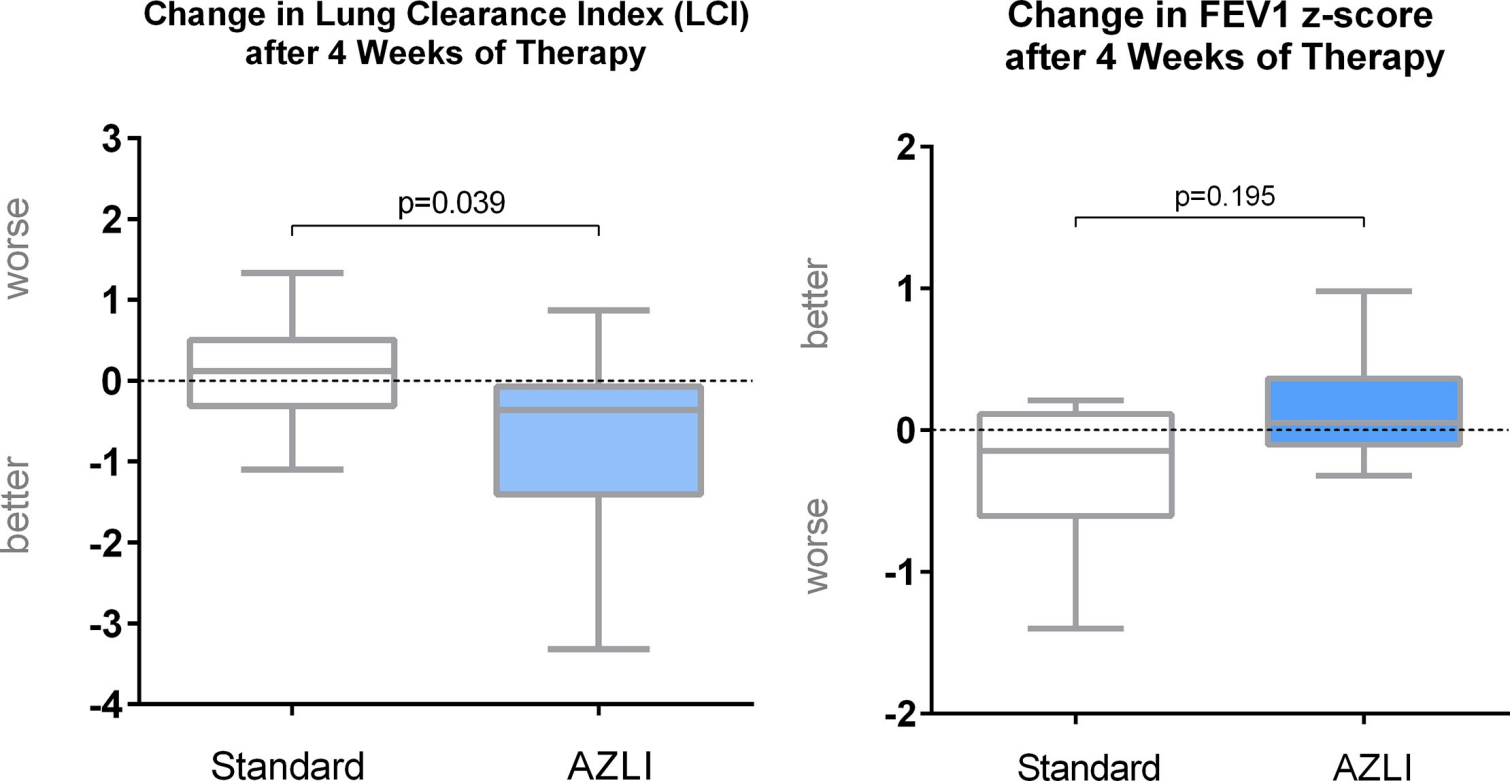
**Changes in LCI (left) or FEV**_**1**_ **z-score (right) after inhaled antibiotics.** More favourable LCI response to AZLI.

The treatment responses determined with LCI were more favourable after AZLI than after tobramycin (-0.365 vs. +0.120, p = 0.039, Fig 3).

In contrast, no statistically significant differences between AZLI and standard treatment were observed with FEV_1_ as an endpoint (FEV_1_ z-score: +0.05 vs -0.15, p = 0.195; FEV_1_% predicted: +0.50 vs -1.80, p = 0.195), S1 and S2 Tables.

Respiratory symptoms showed clinically important improvements after AZLI treatment (median change +8.5) and better results (p = 0.031) than after tobramycin (median -3.0). Treatments were well tolerated, and no adverse events were reported.

## Discussion

In a small group of patients with cystic fibrosis and mild lung disease, greater improvements in lung clearance index and respiratory symptoms were observed after a 4-week course of AZLI compared to the standard inhaled antibiotic tobramycin. In contrast, FEV_1_ did not show significant differences between the two treatment periods. The results of this small feasibility study are in agreement with previous findings that using FEV_1_ as an outcome measure requires larger groups of patients with predominantly moderate to severe lung disease and a greater potential for improvement [15–18].

This is the first comparison of AZLI and tobramycin inhalation in patients with mild lung disease, as indicated by an FEV_1_ > 75% of the predicted normal value. Superiority of AZLI over tobramycin has so far been reported only in patients with more advanced lung disease. After 4 weeks of AZLI inhalation, mean relative improvements of 8.4% from baseline FEV_1_% predicted were observed, while changes after tobramycin (+0.6% form baseline) were considerably smaller [19]. Several studies in relatively healthy patients with mild lung disease have compared AZLI with placebo. In 76 patients with mild lung disease, FEV_1_ improved significantly after AZLI only in patients with a baseline FEV_1_ of < 90% predicted [20]. Also, the AZLI Phase 2 study showed no greater improvement in FEV_1_ after 14 days of AZLI treatment compared to placebo in the subset of 32 patients with FEV_1_ > 75% predicted [1]. Thus, researchers were unable to show greater improvements in lung function after AZLI compared to tobramycin inhalation in mildly affected patients when FEV_1_ was used as the primary outcome parameter [20].

The study had several limitations. First, the patients cohort of this single-centre study was small compared to the international, multi-centre pivotal studies. Second, we were unable to recruit the desired number of 10 patients within a reasonable time. This limits the power of the study.

So far the minimal clinically important difference for LCI has not been defined [18]. Expert committees stated that LCI is a valuable potential outcome parameter in patients with normal FEV_1_, since LCI can detect treatment differences even in small patient groups [15,17].

The results of this feasibility study are in line with the above statements and suggest that LCI should be considered as a clinical endpoint for trials in patients with early lung disease.

## Supporting information

S1 Checklist Consort 2010 checklist. (PDF)

S1 Table FEV1 z score. (XLSX)

S2 Table LCI. (XLSX)

S1 Protocol Clinical trial protocol. (PDF)

## References

[1] Retsch-Bogart GZ, Burns JL, Otto KL, Liou TG, McCoy K, Oermann C et al (2008) A phase 2 study of aztreonam lysine for inhalation to treat patients with cystic fibrosis and Pseudomonas aeruginosa infection. Pediatric pulmonology 43 (1): 47–58. 10.1002/ppul.20736 18041081

[2] Retsch-Bogart GZ, Quittner AL, Gibson RL, Oermann CM, McCoy KS, Montgomery AB et al (2009) Efficacy and safety of inhaled aztreonam lysine for airway pseudomonas in cystic fibrosis. Chest 135 (5): 1223–1232. 10.1378/chest.08-1421 19420195 PMC2818415

[3] Hutchinson D, Barclay M, Prescott WA, Brown J (2013) Inhaled aztreonam lysine. An evidence-based review. Expert opinion on pharmacotherapy 14 (15): 2115–2124. 10.1517/14656566.2013.831070 23992352

[4] O'Neill K, Tunney MM, Johnston E, Rowan S, Downey DG, Rendall J et al (2016) Lung clearance index in adults and children with cystic fibrosis. Chest. 10.1016/j.chest.2016.06.029 27395423

[5] Amin R, Subbarao P, Jabar A, Balkovec S, Jensen R, Kerrigan et al (2010) Hypertonic saline improves the LCI in paediatric patients with CF with normal lung function. Thorax 65 (5): 379–383. 10.1136/thx.2009.125831 20435858

[6] Amin R, Subbarao P, Lou W, Jabar A, Balkovec S, Jensen R et al (2010) The effect of dornase alfa on ventilation inhomogeneity in patients with cystic fibrosis. Eur Respir J. 10.1183/09031936.00072510 20693248

[7] Ellemunter H, Fuchs SI, Unsinn KM, Freund MC, Waltner-Romen M, Steinkamp G et al (2010) Sensitivity of lung clearance index and chest computed tomography in early cf lung disease. Respir Med 104 (12): 1834–1842. 10.1016/j.rmed.2010.06.010 20637585

[8] Lee TW, Brownlee KG, Conway SP, Denton M, Littlewood JM (2003) Evaluation of a new definition for chronic Pseudomonas aeruginosa infection in cystic fibrosis patients. J Cyst.Fibros. 2 (1): 29–34. 10.1016/S1569-1993(02)00141-8 15463843

[9] Fuchs SI, Eder J, Ellemunter H, Gappa M (2009) Lung clearance index: normal values, repeatability, and reproducibility in healthy children and adolescents. Pediatr Pulmonol 44 (12): 1180–1185. 10.1002/ppul.21093 19911370

[10] Quanjer PH, Stanojevic S, Cole TJ, Baur X, Hall GL, Culver BH et al (2012) Multi-ethnic reference values for spirometry for the 3-95-yr age range: the global lung function 2012 equations. Eur Respir J 40 (6): 1324–1343. 10.1183/09031936.00080312 22743675 PMC3786581

[11] Schmidt A, Wenninger K, Niemann N, Wahn U, Staab D (2009) Health-related quality of life in children with cystic fibrosis: validation of the German CFQ-R. Health Qual.Life Outcomes. 7: 97 10.1186/1477-7525-7-97 19954541 PMC2794264

[12] Subbarao P, Stanojevic S, Brown M, Jensen R, Rosenfeld M, Davis et al (2013) Lung clearance index as an outcome measure for clinical trials in young children with cystic fibrosis. A pilot study using inhaled hypertonic saline. Am J Respir Crit Care Med 188 (4): 456–460. 10.1164/rccm.201302-0219OC 23742699 PMC3778738

[13] Konstan MW, Flume PA, Kappler M, Chiron R, Higgins M, Brockhaus F et al (2011) Safety, efficacy and convenience of tobramycin inhalation powder in cystic fibrosis patients. The EAGER trial. Journal of cystic fibrosis: official journal of the European Cystic Fibrosis Society 10 (1): 54–61. 21075062 10.1016/j.jcf.2010.10.003 PMC4086197

[14] Vandevanter DR, Geller DE (2011) Tobramycin administered by the TOBI((R)) Podhaler((R)) for persons with cystic fibrosis. A review. Medical devices (Auckland, N.Z.) 4: 179–188. 10.2147/MDER.S16360 PMC3417888 22915944

[15] Kent L, Reix P, Innes JA, Zielen S, Le Bourgeois M, Braggion C et al (2014) Lung clearance index: Evidence for use in clinical trials in cystic fibrosis. J Cyst Fibros 13 (2): 123–138. 10.1016/j.jcf.2013.09.005 24315208

[16] Ratjen F, Sheridan H, Lee P, Song T, Stone A, Davies JC et al (2011) Lung clearance index as an outcome measure in cystic fibrosis clinical trials [Abstract 201]. Pediatr Pulmonol Suppl.

[17] Subbarao P, Milla C, Aurora P, Davies JC, Davis SD, Hall GL et al (2015) Multiple-Breath Washout as a Lung Function Test in Cystic Fibrosis. A Cystic Fibrosis Foundation Workshop Report. Annals of the American Thoracic Society 12 (6): 932–939. 10.1513/AnnalsATS.201501-021FR 26075554 PMC5466249

[18] Tiddens HAWM, Puderbach M, Venegas JG, Ratjen F, Donaldson SH, Davis SD et al (2015) Novel outcome measures for clinical trials in cystic fibrosis. Pediatric pulmonology 50 (3): 302–315. 10.1002/ppul.23146 25641878 PMC4365726

[19] Assael BM, Pressler T, Bilton D, Fayon M, Fischer R, Chiron R et al (2013) Inhaled aztreonam lysine vs. inhaled tobramycin in cystic fibrosis. A comparative efficacy trial. Journal of cystic fibrosis: official journal of the European Cystic Fibrosis Society 12 (2): 130–140. 22985692 10.1016/j.jcf.2012.07.006

[20] Wainwright CE, Quittner AL, Geller DE, Nakamura C, Wooldridge JL, Gibson RL et al (2011) Aztreonam for inhalation solution (AZLI) in patients with cystic fibrosis, mild lung impairment, and P. aeruginosa. Journal of cystic fibrosis: official journal of the European Cystic Fibrosis Society 10 (4): 234–242. 21441078 10.1016/j.jcf.2011.02.007

