# Supplementary material for: Lung clearance index to detect the efficacy of Aztreonam lysine inhalation in patients with cystic fibrosis and near normal spirometry – A single-centre feasibility study
Source: PLoS One. 2019 Sep 9;14(9):e0221673. doi: 10.1371/journal.pone.0221673 (PMC6733506; doi:10.1371/journal.pone.0221673)
Supplement: S1 Protocol — (PDF) [file pone.0221673.s004.pdf]

LUNG CLEARANCE INDEX AS AN OUTCOME PARAMETER TO DETECT THE EFFICACY OF AZTREONAM LYSINE INHALATION IN CYSTIC FIBROSIS PATIENTS WITH NEAR NORMAL SPIROMETRY – AN OBSERVATIONAL PROOF-OF-CONCEPT STUDY

Proposal for an Investigator Initiated trial
from
Gilead Sciences
and
Study Protocol

Principal Investigator:
Ass. Prof. Dr. med. Helmut Ellemunter, Innsbruck

Version 6, October 01, 2013

Protocol Number	GS V6 03-10-2013
EudraCT-Number	2013-004295-35

Principal Investigator/Coordinating Investigator

Ass. Prof. Dr. med. Helmut Ellemunter
Medizinische Universität Innsbruck
Dept. für Kinder- und Jugendheilkunde
Cystische Fibrose Zentrum
Anichstraße 35
A-6020 Innsbruck
Österreich
Phone: +43 (512) 504 24 902
Fax: +43 (512) 504 67 24 903
E-Mail: helmut.ellemunter@i-med.ac.at

SPONSOR OF THE STUDY

Medical University Innsbruck ,
Department für Kinder- und Jugendheilkunde, Pädiatrie III

PRINCIPAL INVESTIGATOR, STUDY AUTHOR

Ass. Prof. Dr. med. Helmut Ellemunter
Medizinische Universität Innsbruck
Dept. für Kinder- und Jugendheilkunde
Cystische Fibrose Zentrum

The present clinical study, including archiving of study documents, will be conducted according to this protocol, and in compliance with Good Clinical Practices, the Declaration of Helsinki in its latest version, the local laws and regulations and the applicable regulatory requirements.

SIGNATURES

Investigational Medical Product:

Cayston/ Aztreonamlysin

STUDY TITLE:

Lung Clearance Index As An Outcome Parameter To Detect The Efficacy Of Aztreonam Lysine Inhalation In Cystic Fibrosis Patients With Near Normal Spirometry – An Observational Proof-Of-Concept Study

EUDRACT Number: **2013-004295-35**

Declaration of the Sponsor

The present study protocol was subject to critical review. Its content is consistent with the current risk/benefit evaluation of the IMP as well as with the moral, ethical and scientific principles of good clinical practice, the latest version of the Declaration of Helsinki, the local laws and the regulations and the applicable regulatory requirements.

**Sponsor**<br>**Univ.-Prof.Dr.J.I.Stein**	Place, Date, Signature
**Principal Investigator**<br>**Ass.Prof.Dr.H.Ellemunter**	Innsbruck, 3.Oct 2013<br>Place, Date, Signature

The signatories above confirm that they have read this study protocol and agree that it contains all information required for study performance. They also agree to conduct the study as set out in this protocol. It has been understood that all documentation previously not published will be kept in strictest confidence.

SIGNATURES

Investigational Medical Product:

Cayston/ Aztreonamlysin

STUDY TITLE:

Lung Clearance Index As An Outcome Parameter To Detect The Efficacy Of Aztreonam Lysine Inhalation In Cystic Fibrosis Patients With Near Normal Spirometry – An Observational Proof-Of-Concept Study

EUDRACT Number: **2013-004295-35**

Declaration of the Principal (center-specific) Investigator:

I have read this study protocol and agree that it contains all the information required for study performance. I agree to conduct the study as set out in the protocol. In particular, I agree to adhere to the moral, ethical and scientific principles of good clinical practice, the latest version of the declaration of Helsinki, the local laws and regulations and the applicable regulatory requirements.

I understand that all documentation that has not been previously published will be kept in the strictest confidence. This documentation includes the study protocol, investigator's brochure, case report forms, and other scientific data.

Innsbruck, 3.Oct 2013	**Ass.Prof.Dr.H.Ellemunter**
Place, Date	Printed Name
	Signature

1. BACKGROUND

Inhaled antibiotics such as tobramycin, colistin or aztreonam lysine (AZLI, Cayston®) are used to treat chronic *P. aeruginosa* airway infections in patients with CF. The spirometric parameter used most frequently to show the efficacy of respiratory treatments is FEV1, expressed as a percentage of the predicted normal value.

In clinical trials, FEV1 as a primary endpoint requires large numbers of patients to demonstrate the superiority of one treatment over the other. In addition, FEV1 has not proven sensitive enough to detect changes in relatively healthy patients. Since increasing proportions of patients, including adults, nowadays have normal or near normal lung function, better respiratory function parameters that can be used as endpoints in clinical trials are needed.
Multiple breath washout (MBW) technique is a relatively new pulmonary function method which identifies early abnormalities particularly in the small airways, the region of the lung where CF lung disease starts. This technique could be particularly useful to detect treatment induced improvements in CF patients with mild lung disease. Another advantage is that the tests are extremely easy to perform, since they are done during tidal breathing and do not require the co-operation of the patient. This makes the method suitable for small children.

1.1. Multiple Breath Washout technique (MBW)

Each MBW test constitutes of two phases with continuous measurements of gas concentrations and volumes of the exhalations. During the wash-in phase, patients inhale an inert tracer gas mixture during tidal breathing until inspiratory and expiratory concentrations of tracer gas are equal, plus 30 seconds. During the wash-out phase, patients inhale room air, so that each exhalation contains lower concentrations of tracer gas than the preceding exhalation. The wash-out ends when the end-tidal tracer gas concentration is less than $1/40^{th}$ of the starting concentration.

Different instruments and protocols of measuring LCI were published. The authors had participated in the development of the EasyOne Pro LAB MBW Module from ndd Medical Technologies, Zürich, Switzerland. This instrument became commercially available in 2012 and is currently the only MBW device on the market in Europe.
The reliability of the EasyOne Pro LAB has been scientifically proven. The specifications of the sensors are presented on the manufacturer's website (http://www.ndd.ch/Products/easyone-pro-lab.aspx). In healthy volunteers, the within-test repeatability of the lung clearance index was 5.1% [3]. Reproducibility after 1 hour and after > 6 months was 4.2% and 5.1%, respectively, in healthy children and adults [3]. The upper limit of normal was 7.0. The hygienic safety of the instrument has also been published [4]. In a study with cystic fibrosis patients [5] a second MBW measurement was performed after 1 hour, with a mean difference of LCI values of 0.07. Reproducibility was 2.6%. Physiotherapy had no relevant influence on LCI results [5]. A multi-centre evaluation in German CF-units (and Innsbruck) with 183 patients and 151 healthy volunteers revealed a low inter-centre variability of lung clearance index results [1].
Another advantage of the EasyOne Pro LAB compared to other devices is that the nitrogen-washout uses 100% oxygen. No tracer gas is required, while most other

instruments rely on sulphur hexafluoride (SF6), whose global warming potential is 23,900 times greater than that of CO2.

1.2. LCI as an outcome parameter

Two parameters are derived from MBW: the functional residual capacity, FRC, and the lung clearance index.
FRC is calculated as the cumulative expired volume of the tracer gas divided by the difference between the gas concentrations at the start minus those at the end of the measurement.
LCI is defined as the number of lung volume turnovers needed until the lungs are cleared from the inert tracer gas that has been inhaled before. LCI is calculated from the cumulative expired volume divided by the FRC. The resulting value is independent of age, height, or sex, so there is no need to consider normal values, and all patients can be directly compared. LCI values above 7.0 are abnormal and indicate ventilation inhomogeneity. LCI was able to detect early lung disease in patients studied at the CF Centre Innsbruck [2].

2. STUDY PROPOSAL AND PROTOCOL

2.1. Aims of the study

To compare the changes in LCI before and after each 4-week-on/ 4 week-off cycle of different inhaled antibiotics: 1) the patient's standard aerosolised antibiotic (Tobramycin/TOBI 300mg/5ml BID or TOBI Podhaler® 112mg BID, Colistin 2.000.000 I.E. BID) and 2) Aztreonam lysine (AZLI) 75mg TID.
The goal of the study is to show significant improvements in LCI after four weeks of AZLI compared to start of treatment, with each patient serving as his/her own control. We hypothesize that changes after AZLI (phase 2) are not smaller than those during standard antibiotic aerosol (phase 1). LCI measurements are expected to be more change-sensitive than FEV1 measured by spirometry.

2.2. Study Design and Setting

This is an observational, open-label, proof-of-concept study over four months with two treatment phases: one 4-week on/off-cycle with standard (tobramycin or colistin) nebuliser solution followed by one 4-week on/off-cycle with aztreonam lysine (AZLI, Cayston®) nebuliser solution.
For each patient, the study starts at the end of a 4-week period without standard antibiotic aerosol.
The study will be conducted in a single outpatient CF centre, i.e. the CF centre at the University of Innsbruck (for further details see Chapter **3**)
There are five study visits (weeks 0, 4, 8, 12, and 16).

Timeline

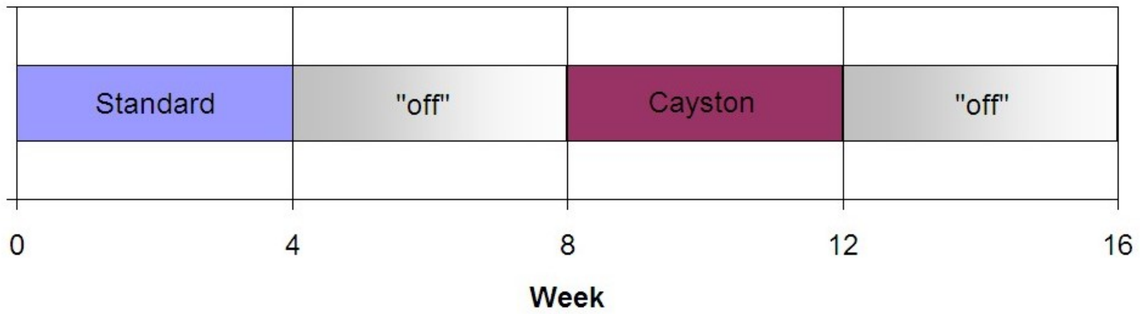

Due to the fact that only few patients in our centre are already being treated with Cayston® as standard treatment, it does not seem feasible to perform a cross-over study. It would be difficult to achieve a consistent starting-point for the treatment periods, and it might be difficult to eliminate other effects.

2.3. Patients and Inclusion/Exclusion Criteria

Inclusion criteria:
Subjects who meet all of the following inclusion criteria will be eligible for this study:

1. Signed informed consent form (ICF), and where appropriate, signed assent form.

2. Males and females, aged 12 years or older on the date of informed consent or, where appropriate, date of assent.

3. Confirmed diagnosis of CF defined as:
    - sweat chloride value ≥60 mmol/L by quantitative pilocarpine iontophoresis
    OR 2 CF-causing mutations (all as documented in the subject's medical record)
    AND
    - Chronic sinopulmonary disease
    OR gastrointestinal/nutritional abnormalities

4. chronic *P. aeruginosa* lung infection

5. clinically stable at outpatient visit

6. FEV1 ≥75% of predicted

7. previous antibiotic aerosol treatment (either tobramycin or colistin, at least 2 on/off cycles or continuous treatment).

Exclusion criteria:
1. Patients with CF related liver disease (CFLD)
    Abnormal liver function defined as any 3 or more of the following: ≥3 × upper limit of normal (ULN) aspartate aminotransferase (AST), ≥3 × ULN alanine aminotransferase (ALT), ≥3 × ULN gamma-glutamyl transpeptidase, ≥3 × ULN alkaline phosphatase, or ≥2 × ULN total bilirubin.

2. Patients with renal disease:
    Abnormal renal function defined as glomerular filtration rate ≤50 mL/min/1.73 m^2 (calculated by the Modification of Diet in Renal Disease Study Equation) for subjects ≥18 years of age and ≤45 mL/min/1.73 m^2 (calculated by the Counahan-Barratt equation) for subjects aged 12 to 17 years (inclusive).

2. Age $\geq$ 50 years

3. An acute upper or lower respiratory infection, pulmonary exacerbation.

4. Pregnant females: Females of childbearing potential must have a negative pregnancy test.

At the CF centre at Innsbruck University in August 2013, 13 patients fulfilled these criteria. Seven subjects are currently treated with inhaled tobramycin only, and the other 6 patients receive antimicrobial rotation with 4 weeks of tobramycin followed by 4 weeks of colistin.

2.4. Study medications

Standard antibiotic aerosol: either tobramycin or colistin solution for inhalation,

- TOBI® 300mg BID or TOBI Podhaler® 112mg BID,
- Colistin® 2.000.00 IU BID;

AZLI: Cayston® 75 mg TID.

2.5. Investigational Medical Product

- Description of medical product (Cayston) is attached to the study protocol

2.6. Drug Accountability

This is a single center study. The product is only supplied to patients in the local hospital and is not used for a multicenter study, this is seen as part of “usual scope of hospital pharmacy” (§ 62 (2) AMG). This means that manufacturing authorization and GMP certificate are not required. CoA und QP certificate of compliance are available.

The investigator or designated site staff will maintain records documenting the dates and amounts of

- study drug received,
- study drug dispensed to the subjects,
- study drug returned by the subjects, and
- study destruction of study drug on site.

Subjects will be instructed to return all used and unused study drug to the site. The study drug will be retained at the site.

Unused study drug is properly destroyed in compliance with all applicable local and national laws, rules, directives, regulations and guidance for any drug that is not distributed under the study, or is remaining due to early termination of the study (as the case may be). Evidence of any such drug destruction is documented in study files and will provided such evidence to Gilead at its request.

2.7. Compliance

To ensure treatment compliance, the investigator or designee will supervise all study drug dosing that occurs at the site. At each visit, site personnel will review that the subject is

compliant with study drug dosing and remind the subject of study drug dosing requirements. Compliance will also be assessed by ongoing study drug count.
If a subject demonstrates continued noncompliance of study drug dosing despite educational efforts, the investigator should discuss discontinuation of the subject from the study.

2.8. Prior and concomitant medications

Information regarding all prior and concomitant medications, including the subject's CF medications, other medications, and herbal and naturopathic remedies administered from 30 days before Visit 1 through Visit 5 will be recorded electronically in the Cystic Fibrosis Centre database and case report form (CRF).

2.9. Treatment

Phase 1: Four weeks of standard antibiotic inhalation ("on", weeks 0 to 4), followed by four weeks without antibiotic inhalation ("off", weeks 4 to 8)
Phase 2: Four weeks of AZLI inhalation ("on", weeks 8 to 12), followed by four weeks without AZLI ("off", weeks 12 to 16).

2.10. Measurements

- Multiple Breath Washout with Lung Clearance Index (LCI)
- Spirometry
- Respiratory scale of the CFQ-R quality of life questionnaire
- Adverse events

As part of routine monitoring:

- Sputum sample/throat swabs for bacterial culture
- Blood samples: Complete blood count (CBC), immunoglobulin G, C-reactive protein (CRP)

Visit Plan

	Visit 1	Visit 2	Visit 3	Visit 4	Visit 5
**Week**	**0**	**4**	**8**	**12**	**16**
Outpatient visit (history and physical examination, weight and length)	x	x	x	x	x
Adverse events		x	x	x	x
Microbiology	x	x	x	x	x
Clinical chemistry	x	x	x	x	x
CFQ-R respiratory scale	x	x	x	x	x
Spirometry	x	x	x	x	x
Multiple breath washout (MBW)	x	x	x	x	x

2.11. Primary endpoint

Comparison of the relative changes in LCI (Delta LCI) after treatment with AZLI and after standard therapy:
Computation of the change in LCI during treatment (Delta LCI) for

- AZLI: the difference in LCI after AZLI inhalation (at week 12) compared to base-line before AZLI (at week 8) and for
- standard treatment: the difference in LCI after standard treatment (at week 4) compared to base-line (at week 0). Different standard treatments will be analysed separately, and if treatment responses are comparable, a pooled analysis will be performed.

2.12. Other endpoints

Comparison of the relative changes in FEV1 (Delta FEV1) after treatments with AZLI and after standard therapy (computation as described for the primary endpoint)
Changes in LCI and FEV1 during the off-periods after AZLI (week 16 compared to week 12) and after standard antibiotic aerosol (week 8 compared to week 4).

2.13. Estimated sample size

To estimate the appropriate sample size, it is crucial to consider the relation between standard deviation and the difference after treatment for the primary endpoint. During the last five years we performed numerous LCI measurements in our patient cohort and found a LCI standard deviation of 1.25 in a rather healthy subgroup of patients. The mean change in LCI after treatment with hypertonic saline was 0.74. A research group from Canada recently reported a change in LCI of 2.2 after treatment with Ivacaftor (Davies et al, ECFC 2012).

We assume that LCI will improve after Cayston by at least the same magnitude as after hypertonic saline, probably more. Using the above mentioned figures, a sample size of 10

/ 12 has a 80% power to detect a difference between means of 0.85 / 0.77 with a significance level (alpha) of 0.05 (two-tailed) in a paired t-test.

Thus, it seems reasonable to undertake the proposed trial with Cayston, although only 13 patients are available for study at our centre.

2.14. Documentation

Subject and disease characteristics, medical history, actual therapy/concomitant medication is stored electronically in the Cystic Fibrosis Centre database. Study specific data are documented in the CRF each visit. All AEs will be collected from the time informed consent is signed.

2.15. Monitoring

Monitoring procedures developed or endorsed by the Sponsor will be adhered to, in order to comply with ICH-GCP guidelines and local legal requirements to ensure acceptability of the study data.

Study Initiation and Monitoring
The Investigator must grant direct access to on-site study documentation, including the patients notes, to allow audits or inspections to be performed. No action will be taken that might infringe the patients confidentiality.

Monitoring visits by representatives of the Sponsor will be carried out to review study plan compliance, to compare CRFs and individual patient medical records, to perform accounting of study material, and to ensure that the study is being conducted according to pertinent regulatory requirements.
CRF entries will be verified with source documentation. The frequency and duration of Monitoring visits will be determined according to clinical site accrual, site performance, adherence to the protocol, and data quality.

Further functions of the Monitor are:
-Assessment, if the Clinical Trial Center is in accordance with the demands of the clinical study (patient population, equipment, storage of the study material etc.)
-Instruction of the Investigators and study staff in the clinical study
-Inspection of the Investigator Site File (ISF)
-Documentation of the patient status
-CRF-data verification with the source data
-Evaluation of the SAEs reports according to the regulations
-Evaluation of the proper and secure storage as well as the storage life of the investigational medicinal product
-Counting of the returned investigational medicinal product (Drug accountability), evaluation of the compliance

3. THE CF CENTRE AT INNSBRUCK UNIVERSITY

The CF centre Innsbruck cares for 167 patients of all age groups. It works according to high quality standards, and the centre has received repeated ISO 9001:2008 certificates. A patient database was established in 1995 which contains data of all outpatient encounters. Graphs and tables displaying important clinical and lung function results are used during outpatient clinic visits and are shown to patients and parents to support the mutual discussion of the course of the disease.

The Principle Investigator of the proposed study, Prof. Dr. med. Helmut Ellemunter, has been serving as the head of the Innsbruck CF centre for the last 27 years. Other personnel have shown remarkably low fluctuation rates, so that the centre has exceptionally experienced staff.

The CF centre has participated in several multi-centre clinical trials. In particular, a co-operation with Prof. Dr. Monika Gappa and Dr. Susanne Fuchs (Wesel and Hannover) was established in 2007 to compare MBW measurements and high resolution CTs in mildly affected patients with CF (see ref 2). During the last five years, the CF centre has continued to measure LCI repeatedly in patients with near normal lung function, so that more than 350 measurements have meanwhile been recorded.

4. REFERENCES

1) Fuchs SI, Ellemunter H, Eder J, Mellies U, Grosse-Onnebrink J, Tümmler B, Staab D, Jobst A, Griese M, Ripper J, Rietschel E, Zeidler S, Ahrens F, Gappa M. Feasibility and Variability of Measuring the Lung Clearance Index in a Multi-Center Setting. Pediatr Pulmonol. 2012 Jul;47(7):649-57.

2) Ellemunter H, Fuchs SI, Unsinn KM, Freund MC, Waltner-Romen M, Steinkamp G, Gappa M. Sensitivity of lung clearance index and chest computed tomography in early CF lung disease. Respir Med 2010 Dec;104(12):1834-42.

3) Fuchs SI, Eder J, Ellemunter H, Gappa M. Lung clearance index: Normal values, repeatability, and reproducibility in healthy children and adolescents. Pediatr Pulmonol 2009 Dec;44(12):1180-85.

4) Fuchs SI, Gappa M, Waltner-Romen M, Ellemunter H. Hygienic safety of an ultrasonic flow sensor for multiple breath washout. Pediatr Pulmonol 2009 Jan; 44(1):99-100.

5) Fuchs SI, Toussaint S, Edlhaimb B, Ballmann M, Gappa M. Short-term effect of physiotherapy on variability of the lung clearance index in children with cystic fibrosis. *Pediatr Pulmonol* 45 (3):301-306, 2010.

6) Ellemunter H, Eder J, and Steinkamp G. Strukturierte Versorgung von Mukoviszidosepatienten und ihren Angehörigen in einem ISO-zertifizierten Behandlungszentrum. [Structured Care in an ISO Certified Centre for Patients With Cystic Fibrosis and Their Families.]. Pneumologie 65:615-623, 2011.
